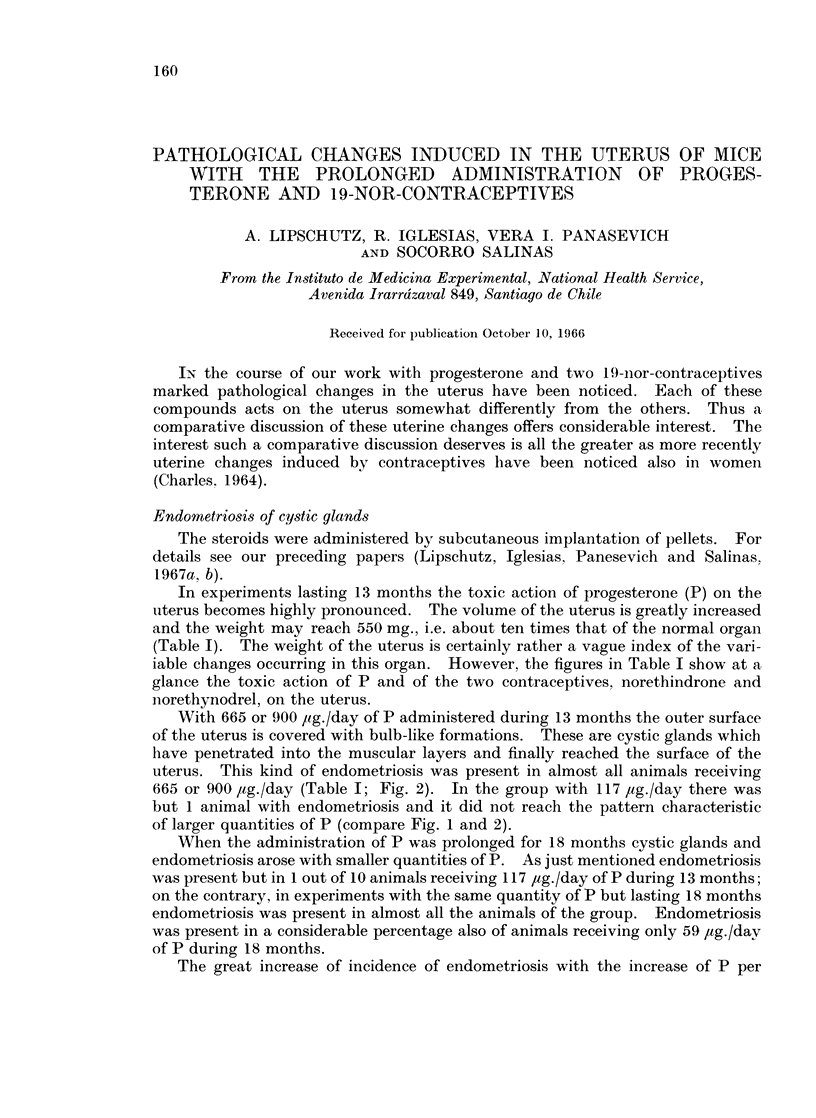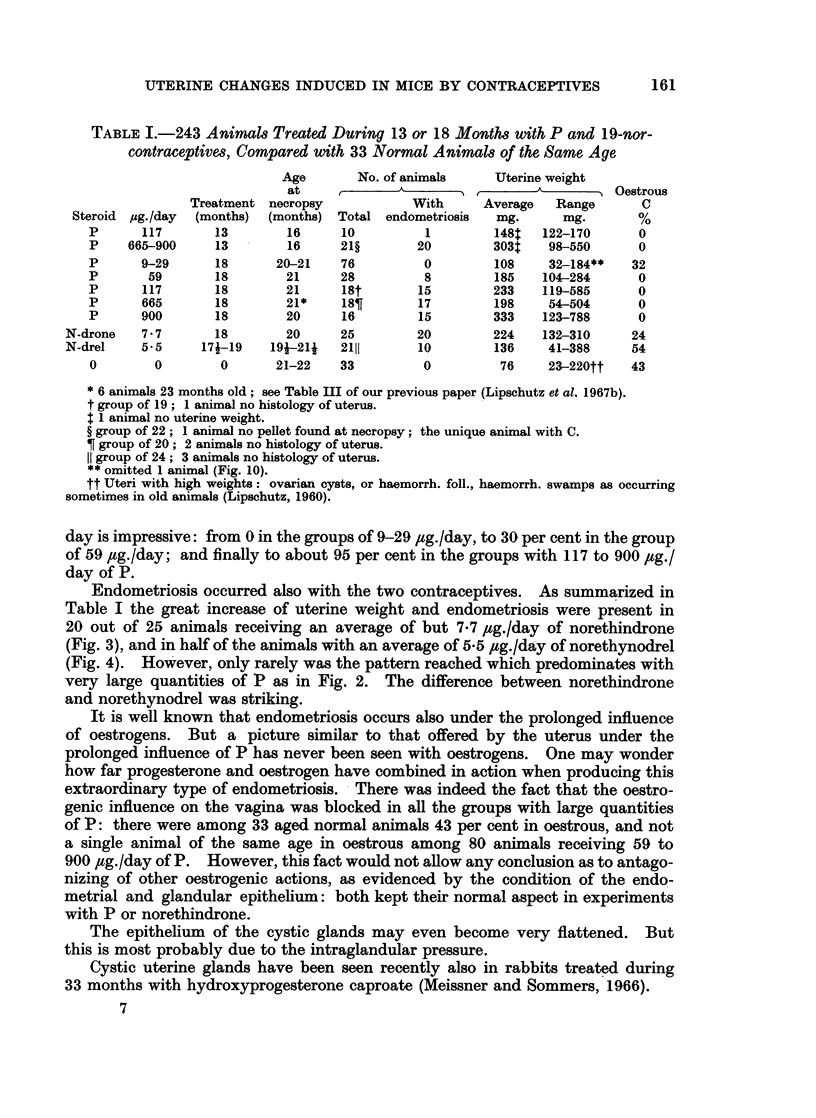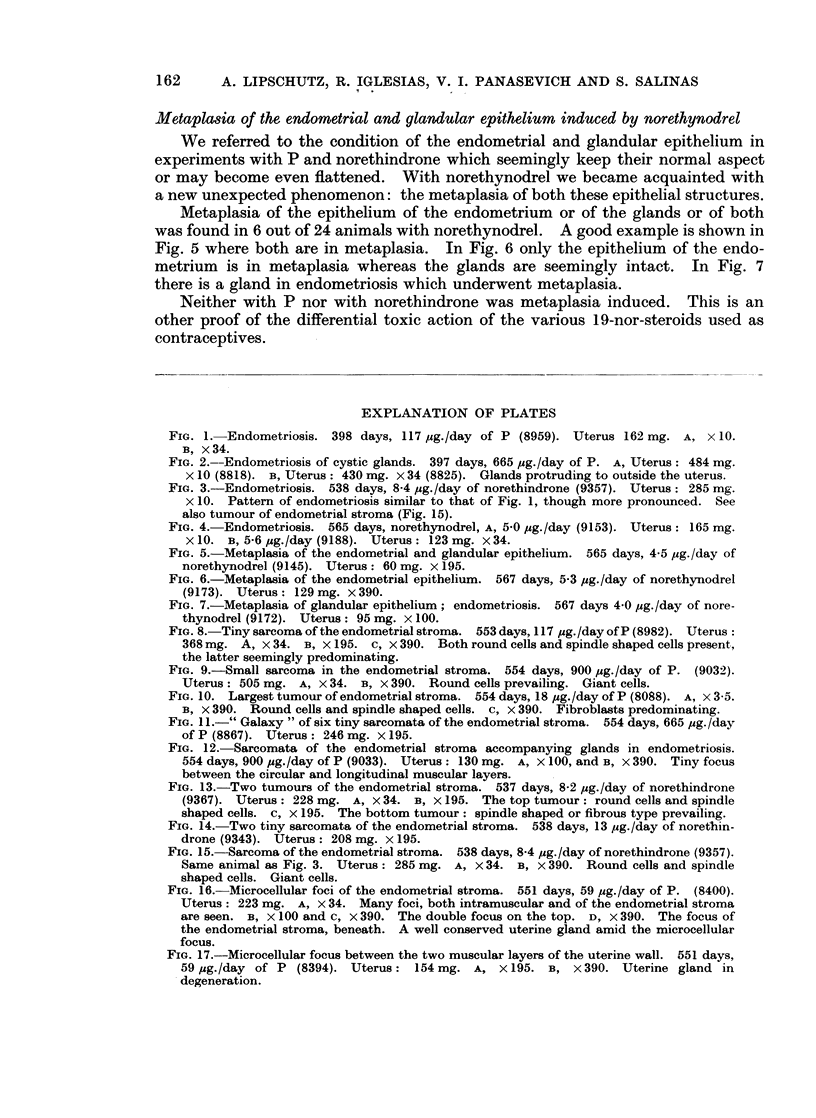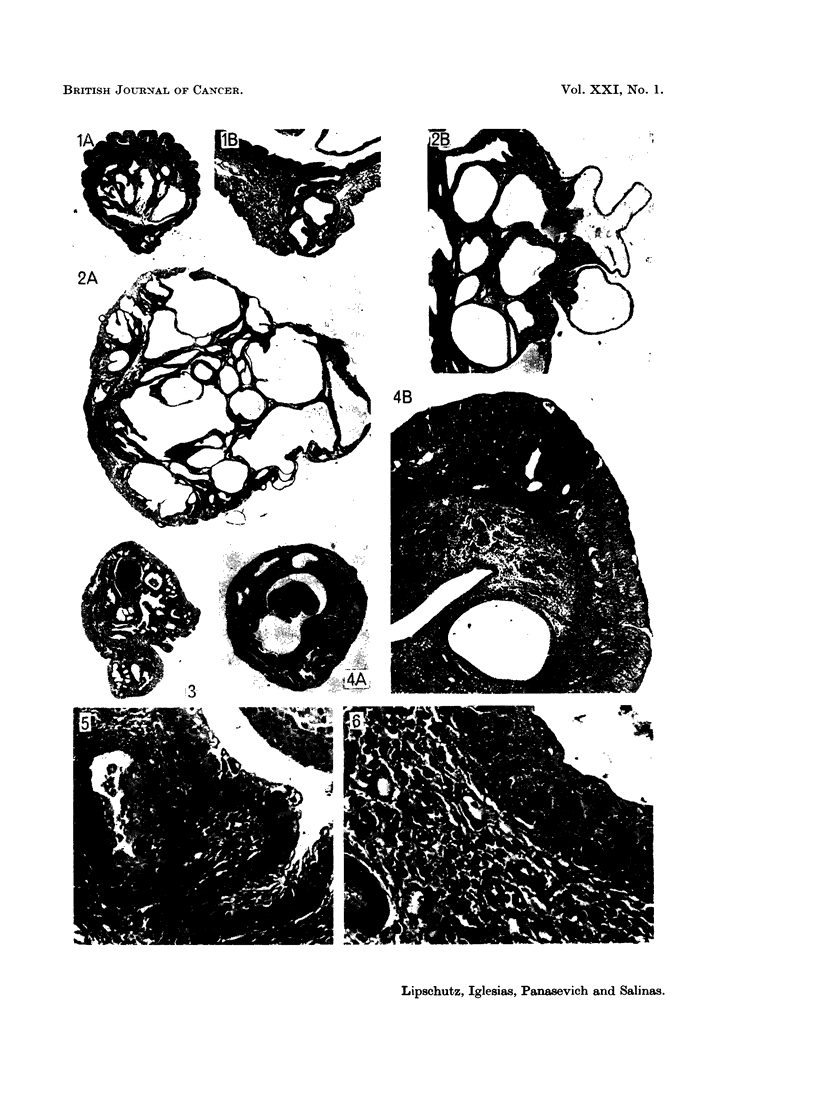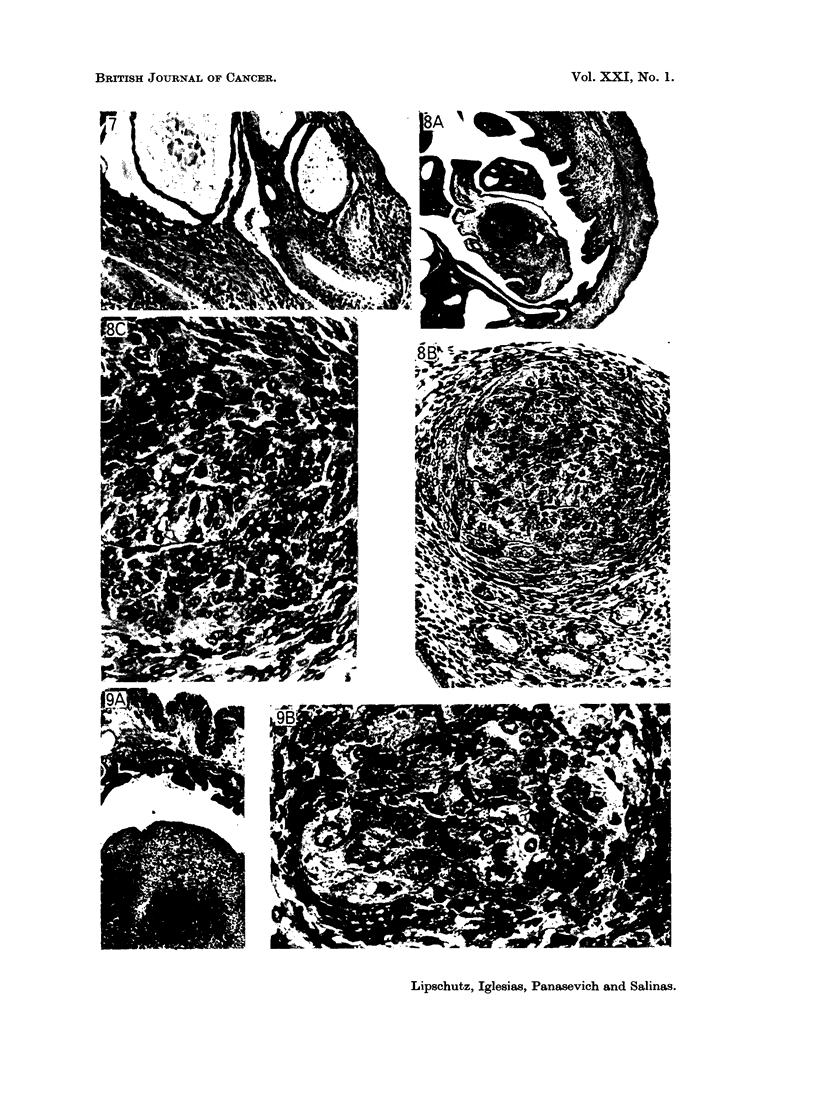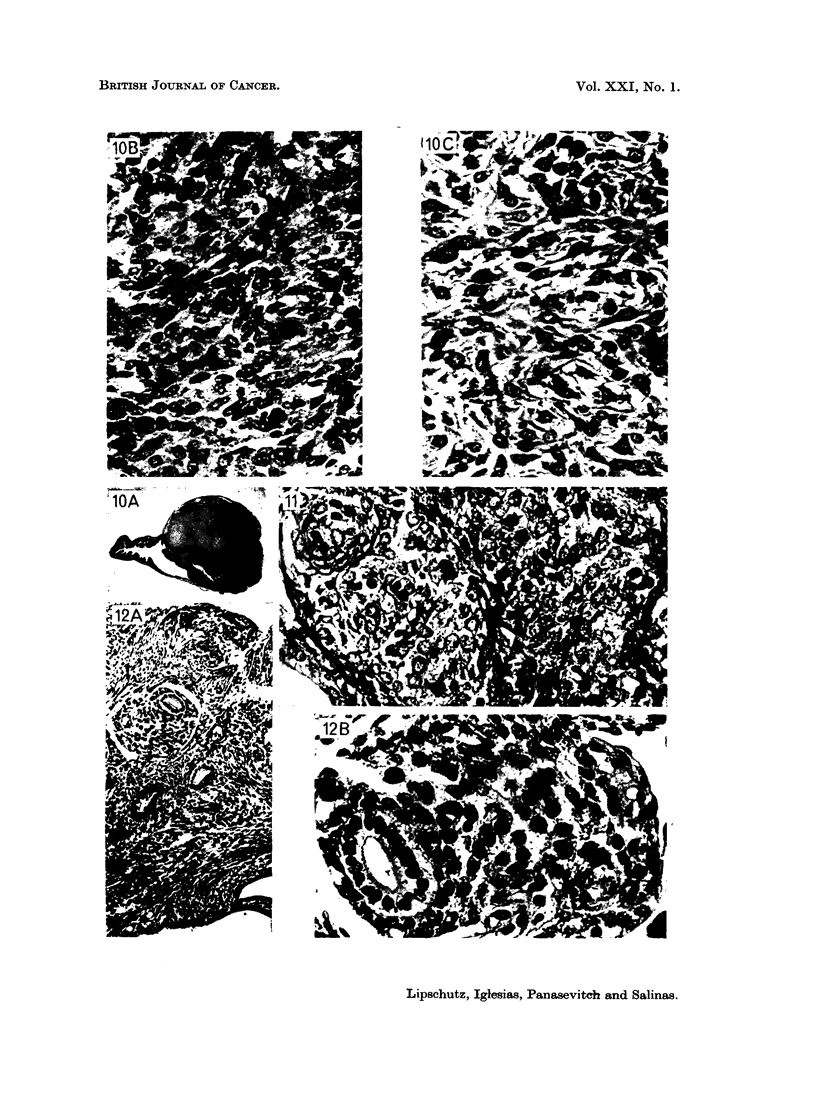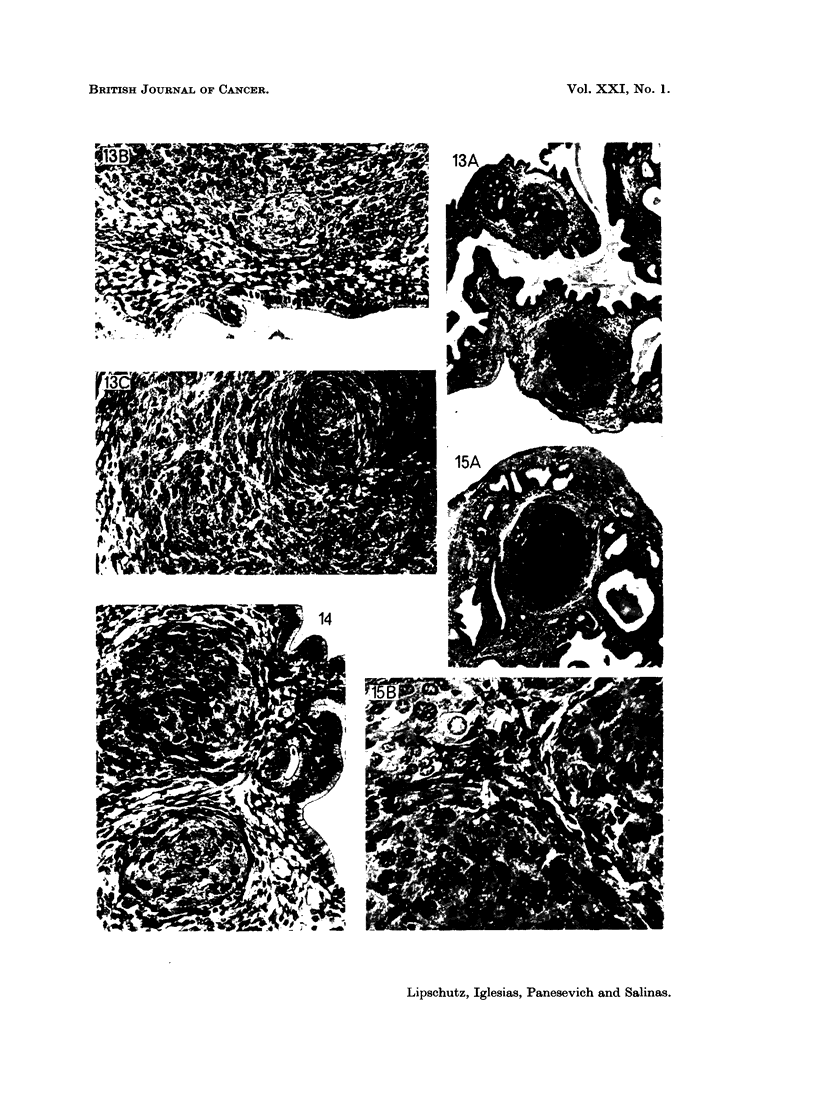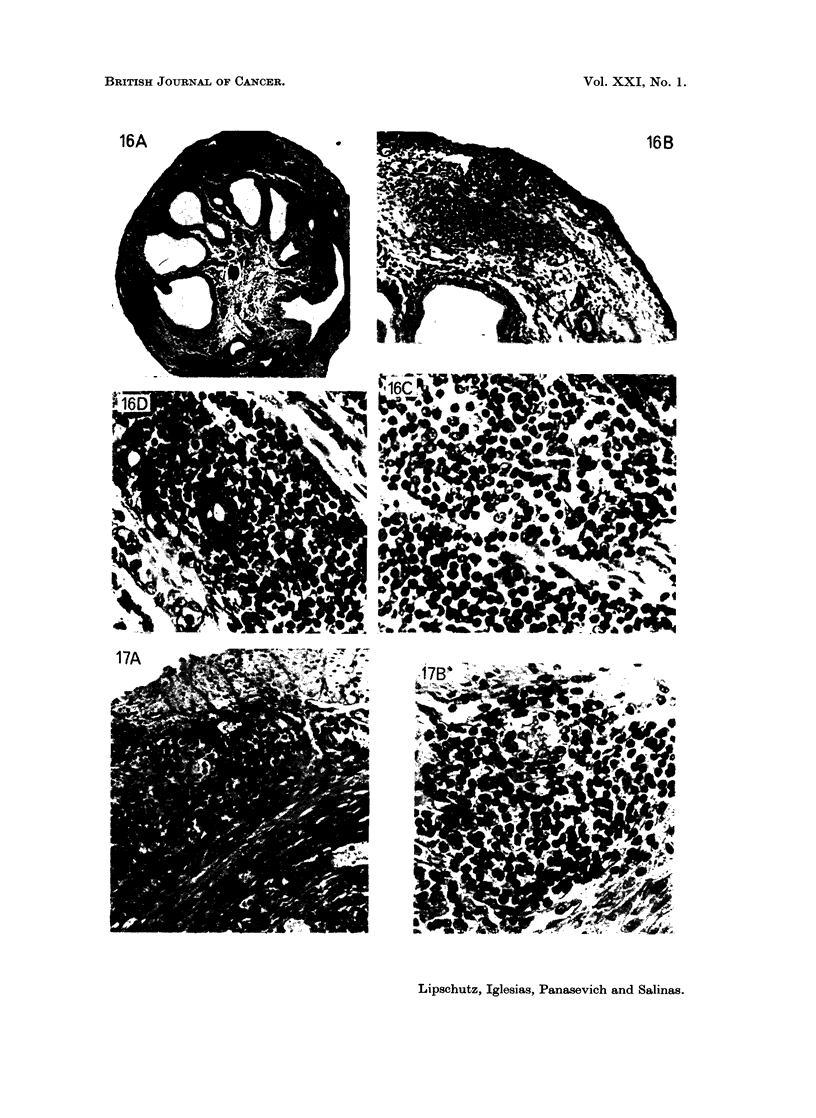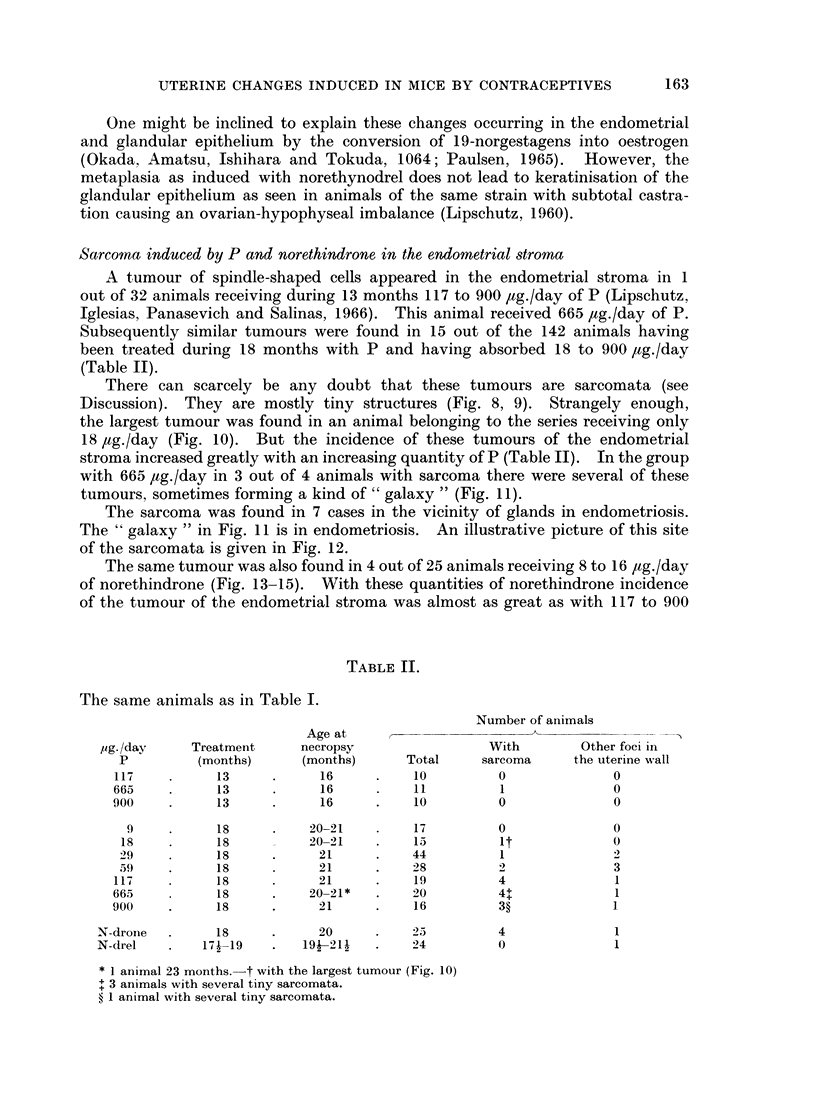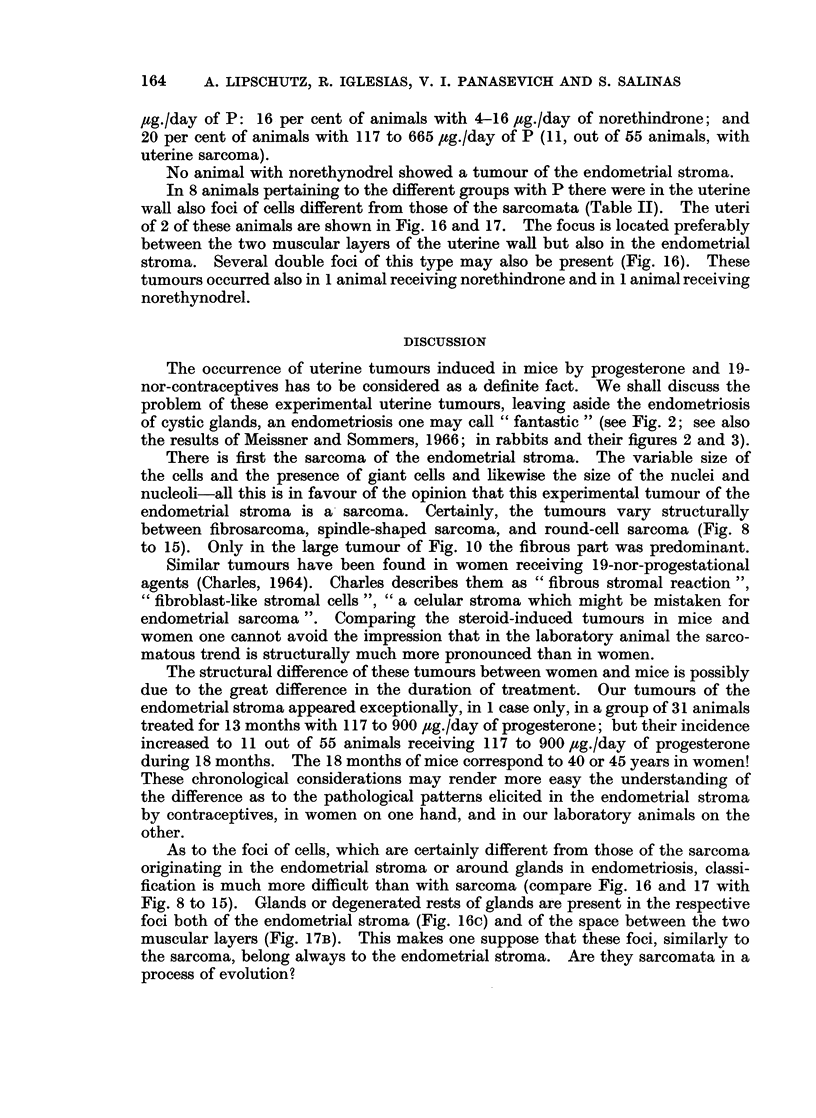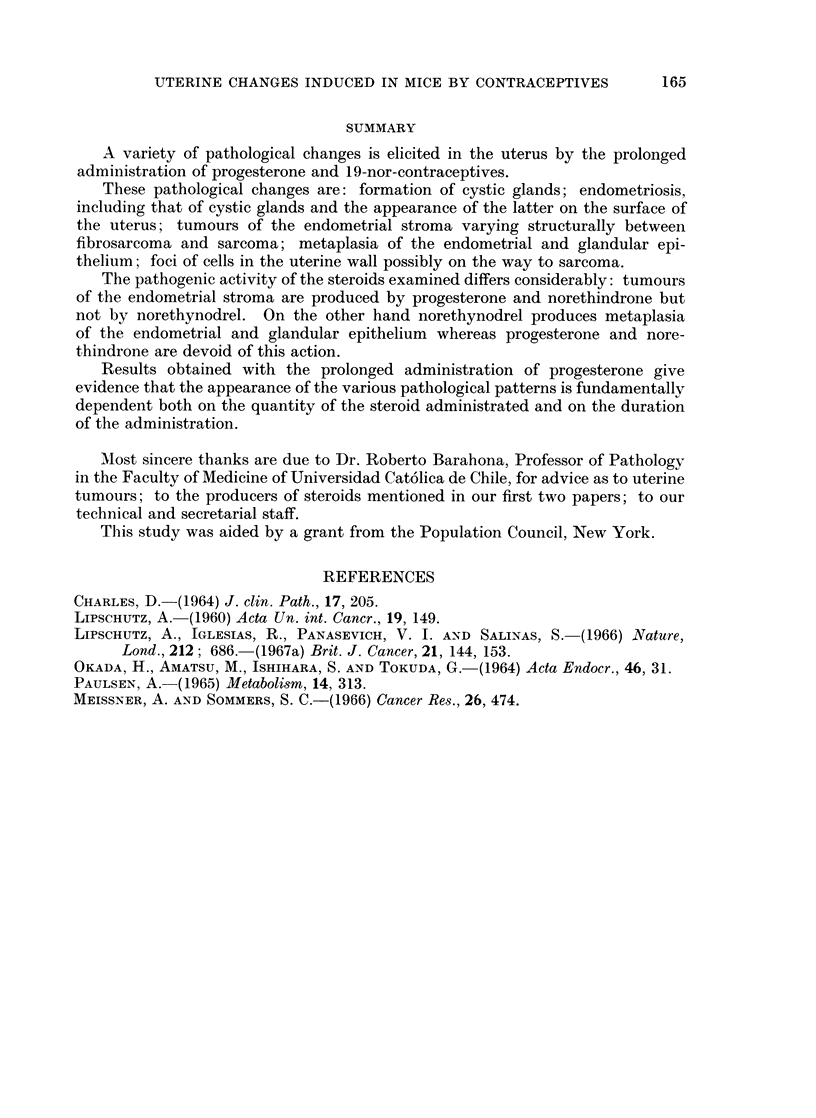# Pathological changes induced in the uterus of mice with the prolonged administration of progesterone and 19-nor-contraceptives.

**DOI:** 10.1038/bjc.1967.15

**Published:** 1967-03

**Authors:** A. Lipschutz, R. Iglesias, V. I. Panasevich, S. Salinas

## Abstract

**Images:**


### PATHOLOGICAL CHANGES INDUCED IN THE UTERUS OF MICE WITH THE PROLONGED ADMINISTRATION OF PROGESTERONE AND 19-NOR-CONTRACEPTIVES

A. LIPSCHUTZ, R. IGLESIAS, VERA I. PANASEVICH AND SOCORRO SALINAS

*From the Instituto de Medicina Experimental, National Health Service, Avenida Irarrázaval 849, Santiago de Chile*

Received for publication October 10, 1966

In the course of our work with progesterone and two 19-nor-contraceptives marked pathological changes in the uterus have been noticed. Each of these compounds acts on the uterus somewhat differently from the others. Thus a comparative discussion of these uterine changes offers considerable interest. The interest such a comparative discussion deserves is all the greater as more recently uterine changes induced by contraceptives have been noticed also in women (Charles, 1964).

*Endometriosis of cystic glands*

The steroids were administered by subcutaneous implantation of pellets. For details see our preceding papers (Lipschutz, Iglesias, Panesevich and Salinas, 1967*a*, *b*).

In experiments lasting 13 months the toxic action of progesterone (P) on the uterus becomes highly pronounced. The volume of the uterus is greatly increased and the weight may reach 550 mg., i.e. about ten times that of the normal organ (Table I). The weight of the uterus is certainly rather a vague index of the variable changes occurring in this organ. However, the figures in Table I show at a glance the toxic action of P and of the two contraceptives, norethindrone and norethynodrel, on the uterus.

With 665 or 900 $\mu$g./day of P administered during 13 months the outer surface of the uterus is covered with bulb-like formations. These are cystic glands which have penetrated into the muscular layers and finally reached the surface of the uterus. This kind of endometriosis was present in almost all animals receiving 665 or 900 $\mu$g./day (Table I; Fig. 2). In the group with 117 $\mu$g./day there was but 1 animal with endometriosis and it did not reach the pattern characteristic of larger quantities of P (compare Fig. 1 and 2).

When the administration of P was prolonged for 18 months cystic glands and endometriosis arose with smaller quantities of P. As just mentioned endometriosis was present but in 1 out of 10 animals receiving 117 $\mu$g./day of P during 13 months; on the contrary, in experiments with the same quantity of P but lasting 18 months endometriosis was present in almost all the animals of the group. Endometriosis was present in a considerable percentage also of animals receiving only 59 $\mu$g./day of P during 18 months.

The great increase of incidence of endometriosis with the increase of P per

TABLE I.—243 *Animals Treated During* 13 *or* 18 *Months with P and* 19-*nor-contraceptives, Compared with* 33 *Normal Animals of the Same Age*

| Steroid | µg./day | Treatment (months) | Age at necropsy (months) | No. of animals: Total | No. of animals: With endometriosis | Uterine weight: Average mg. | Uterine weight: Range mg. | Oestrous C % |
|---|---|---|---|---|---|---|---|---|
| P | 117 | 13 | 16 | 10 | 1 | 148‡ | 122–170 | 0 |
| P | 665–900 | 13 | 16 | 21§ | 20 | 303‡ | 98–550 | 0 |
| P | 9–29 | 18 | 20–21 | 76 | 0 | 108 | 32–184** | 32 |
| P | 59 | 18 | 21 | 28 | 8 | 185 | 104–284 | 0 |
| P | 117 | 18 | 21 | 18† | 15 | 233 | 119–585 | 0 |
| P | 665 | 18 | 21* | 18¶ | 17 | 198 | 54–504 | 0 |
| P | 900 | 18 | 20 | 16 | 15 | 333 | 123–788 | 0 |
| N-drone | 7·7 | 18 | 20 | 25 | 20 | 224 | 132–310 | 24 |
| N-drel | 5·5 | 17½–19 | 19½–21½ | 21‖ | 10 | 136 | 41–388 | 54 |
| 0 | 0 | 0 | 21–22 | 33 | 0 | 76 | 23–220†† | 43 |

* 6 animals 23 months old ; see Table III of our previous paper (Lipschutz *et al.* 1967b).
† group of 19 ; 1 animal no histology of uterus.
‡ 1 animal no uterine weight.
§ group of 22 ; 1 animal no pellet found at necropsy ; the unique animal with C.
¶ group of 20 ; 2 animals no histology of uterus.
‖ group of 24 ; 3 animals no histology of uterus.
** omitted 1 animal (Fig. 10).
†† Uteri with high weights : ovarian cysts, or haemorrh. foll., haemorrh. swamps as occurring sometimes in old animals (Lipschutz, 1960).

day is impressive: from 0 in the groups of 9–29 µg./day, to 30 per cent in the group of 59 µg./day; and finally to about 95 per cent in the groups with 117 to 900 µg./day of P.

Endometriosis occurred also with the two contraceptives. As summarized in Table I the great increase of uterine weight and endometriosis were present in 20 out of 25 animals receiving an average of but 7·7 µg./day of norethindrone (Fig. 3), and in half of the animals with an average of 5·5 µg./day of norethynodrel (Fig. 4). However, only rarely was the pattern reached which predominates with very large quantities of P as in Fig. 2. The difference between norethindrone and norethynodrel was striking.

It is well known that endometriosis occurs also under the prolonged influence of oestrogens. But a picture similar to that offered by the uterus under the prolonged influence of P has never been seen with oestrogens. One may wonder how far progesterone and oestrogen have combined in action when producing this extraordinary type of endometriosis. There was indeed the fact that the oestrogenic influence on the vagina was blocked in all the groups with large quantities of P: there were among 33 aged normal animals 43 per cent in oestrous, and not a single animal of the same age in oestrous among 80 animals receiving 59 to 900 µg./day of P. However, this fact would not allow any conclusion as to antagonizing of other oestrogenic actions, as evidenced by the condition of the endometrial and glandular epithelium: both kept their normal aspect in experiments with P or norethindrone.

The epithelium of the cystic glands may even become very flattened. But this is most probably due to the intraglandular pressure.

Cystic uterine glands have been seen recently also in rabbits treated during 33 months with hydroxyprogesterone caproate (Meissner and Sommers, 1966).

*Metaplasia of the endometrial and glandular epithelium induced by norethynodrel*

We referred to the condition of the endometrial and glandular epithelium in experiments with P and norethindrone which seemingly keep their normal aspect or may become even flattened. With norethynodrel we became acquainted with a new unexpected phenomenon: the metaplasia of both these epithelial structures.

Metaplasia of the epithelium of the endometrium or of the glands or of both was found in 6 out of 24 animals with norethynodrel. A good example is shown in Fig. 5 where both are in metaplasia. In Fig. 6 only the epithelium of the endometrium is in metaplasia whereas the glands are seemingly intact. In Fig. 7 there is a gland in endometriosis which underwent metaplasia.

Neither with P nor with norethindrone was metaplasia induced. This is an other proof of the differential toxic action of the various 19-nor-steroids used as contraceptives.

---

EXPLANATION OF PLATES

FIG. 1.—Endometriosis. 398 days, 117 μg./day of P (8959). Uterus 162 mg. A, ×10. B, ×34.

FIG. 2.—Endometriosis of cystic glands. 397 days, 665 μg./day of P. A, Uterus: 484 mg. ×10 (8818). B, Uterus: 430 mg. ×34 (8825). Glands protruding to outside the uterus.

FIG. 3.—Endometriosis. 538 days, 8·4 μg./day of norethindrone (9357). Uterus: 285 mg. ×10. Pattern of endometriosis similar to that of Fig. 1, though more pronounced. See also tumour of endometrial stroma (Fig. 15).

FIG. 4.—Endometriosis. 565 days, norethynodrel, A, 5·0 μg./day (9153). Uterus: 165 mg. ×10. B, 5·6 μg./day (9188). Uterus: 123 mg. ×34.

FIG. 5.—Metaplasia of the endometrial and glandular epithelium. 565 days, 4·5 μg./day of norethynodrel (9145). Uterus: 60 mg. ×195.

FIG. 6.—Metaplasia of the endometrial epithelium. 567 days, 5·3 μg./day of norethynodrel (9173). Uterus: 129 mg. ×390.

FIG. 7.—Metaplasia of glandular epithelium; endometriosis. 567 days 4·0 μg./day of norethynodrel (9172). Uterus: 95 mg. ×100.

FIG. 8.—Tiny sarcoma of the endometrial stroma. 553 days, 117 μg./day of P (8982). Uterus: 368 mg. A, ×34. B, ×195. C, ×390. Both round cells and spindle shaped cells present, the latter seemingly predominating.

FIG. 9.—Small sarcoma in the endometrial stroma. 554 days, 900 μg./day of P. (9032). Uterus: 505 mg. A, ×34. B, ×390. Round cells prevailing. Giant cells.

FIG. 10. Largest tumour of endometrial stroma. 554 days, 18 μg./day of P (8088). A, ×3·5. B, ×390. Round cells and spindle shaped cells. C, ×390. Fibroblasts predominating.

FIG. 11.—" Galaxy " of six tiny sarcomata of the endometrial stroma. 554 days, 665 μg./day of P (8867). Uterus: 246 mg. ×195.

FIG. 12.—Sarcomata of the endometrial stroma accompanying glands in endometriosis. 554 days, 900 μg./day of P (9033). Uterus: 130 mg. A, ×100, and B, ×390. Tiny focus between the circular and longitudinal muscular layers.

FIG. 13.—Two tumours of the endometrial stroma. 537 days, 8·2 μg./day of norethindrone (9367). Uterus: 228 mg. A, ×34. B, ×195. The top tumour: round cells and spindle shaped cells. C, ×195. The bottom tumour: spindle shaped or fibrous type prevailing.

FIG. 14.—Two tiny sarcomata of the endometrial stroma. 538 days, 13 μg./day of norethindrone (9343). Uterus: 208 mg. ×195.

FIG. 15.—Sarcoma of the endometrial stroma. 538 days, 8·4 μg./day of norethindrone (9357). Same animal as Fig. 3. Uterus: 285 mg. A, ×34. B, ×390. Round cells and spindle shaped cells. Giant cells.

FIG. 16.—Microcellular foci of the endometrial stroma. 551 days, 59 μg./day of P. (8400). Uterus: 223 mg. A, ×34. Many foci, both intramuscular and of the endometrial stroma are seen. B, ×100 and C, ×390. The double focus on the top. D, ×390. The focus of the endometrial stroma, beneath. A well conserved uterine gland amid the microcellular focus.

FIG. 17.—Microcellular focus between the two muscular layers of the uterine wall. 551 days, 59 μg./day of P (8394). Uterus: 154 mg. A, ×195. B, ×390. Uterine gland in degeneration.

Lipschutz, Iglesias, Panasevich and Salinas.

Lipschutz, Iglesias, Panasevich and Salinas.

10B
10C
10A
11
12A
12B

Lipschutz, Iglesias, Panesevich and Salinas.

Lipschutz, Iglesias, Panasevich and Salinas.

One might be inclined to explain these changes occurring in the endometrial and glandular epithelium by the conversion of 19-norgestagens into oestrogen (Okada, Amatsu, Ishihara and Tokuda, 1064; Paulsen, 1965). However, the metaplasia as induced with norethynodrel does not lead to keratinisation of the glandular epithelium as seen in animals of the same strain with subtotal castration causing an ovarian-hypophyseal imbalance (Lipschutz, 1960).

*Sarcoma induced by P and norethindrone in the endometrial stroma*

A tumour of spindle-shaped cells appeared in the endometrial stroma in 1 out of 32 animals receiving during 13 months 117 to 900 μg./day of P (Lipschutz, Iglesias, Panasevich and Salinas, 1966). This animal received 665 μg./day of P. Subsequently similar tumours were found in 15 out of the 142 animals having been treated during 18 months with P and having absorbed 18 to 900 μg./day (Table II).

There can scarcely be any doubt that these tumours are sarcomata (see Discussion). They are mostly tiny structures (Fig. 8, 9). Strangely enough, the largest tumour was found in an animal belonging to the series receiving only 18 μg./day (Fig. 10). But the incidence of these tumours of the endometrial stroma increased greatly with an increasing quantity of P (Table II). In the group with 665 μg./day in 3 out of 4 animals with sarcoma there were several of these tumours, sometimes forming a kind of " galaxy " (Fig. 11).

The sarcoma was found in 7 cases in the vicinity of glands in endometriosis. The " galaxy " in Fig. 11 is in endometriosis. An illustrative picture of this site of the sarcomata is given in Fig. 12.

The same tumour was also found in 4 out of 25 animals receiving 8 to 16 μg./day of norethindrone (Fig. 13–15). With these quantities of norethindrone incidence of the tumour of the endometrial stroma was almost as great as with 117 to 900

TABLE II.

The same animals as in Table I.

| μg./day P | Treatment (months) | Age at necropsy (months) | Number of animals: Total | With sarcoma | Other foci in the uterine wall |
|---|---|---|---|---|---|
| 117 | 13 | 16 | 10 | 0 | 0 |
| 665 | 13 | 16 | 11 | 1 | 0 |
| 900 | 13 | 16 | 10 | 0 | 0 |
| 9 | 18 | 20–21 | 17 | 0 | 0 |
| 18 | 18 | 20–21 | 15 | 1† | 0 |
| 29 | 18 | 21 | 44 | 1 | 2 |
| 59 | 18 | 21 | 28 | 2 | 3 |
| 117 | 18 | 21 | 19 | 4 | 1 |
| 665 | 18 | 20–21* | 20 | 4‡ | 1 |
| 900 | 18 | 21 | 16 | 3§ | 1 |
| N-drone | 18 | 20 | 25 | 4 | 1 |
| N-drel | 17½–19 | 19½–21½ | 24 | 0 | 1 |

* 1 animal 23 months.—† with the largest tumour (Fig. 10)
‡ 3 animals with several tiny sarcomata.
§ 1 animal with several tiny sarcomata.

μg./day of P: 16 per cent of animals with 4–16 μg./day of norethindrone; and 20 per cent of animals with 117 to 665 μg./day of P (11, out of 55 animals, with uterine sarcoma).

No animal with norethynodrel showed a tumour of the endometrial stroma.

In 8 animals pertaining to the different groups with P there were in the uterine wall also foci of cells different from those of the sarcomata (Table II). The uteri of 2 of these animals are shown in Fig. 16 and 17. The focus is located preferably between the two muscular layers of the uterine wall but also in the endometrial stroma. Several double foci of this type may also be present (Fig. 16). These tumours occurred also in 1 animal receiving norethindrone and in 1 animal receiving norethynodrel.

### DISCUSSION

The occurrence of uterine tumours induced in mice by progesterone and 19-nor-contraceptives has to be considered as a definite fact. We shall discuss the problem of these experimental uterine tumours, leaving aside the endometriosis of cystic glands, an endometriosis one may call " fantastic " (see Fig. 2; see also the results of Meissner and Sommers, 1966; in rabbits and their figures 2 and 3).

There is first the sarcoma of the endometrial stroma. The variable size of the cells and the presence of giant cells and likewise the size of the nuclei and nucleoli—all this is in favour of the opinion that this experimental tumour of the endometrial stroma is a sarcoma. Certainly, the tumours vary structurally between fibrosarcoma, spindle-shaped sarcoma, and round-cell sarcoma (Fig. 8 to 15). Only in the large tumour of Fig. 10 the fibrous part was predominant.

Similar tumours have been found in women receiving 19-nor-progestational agents (Charles, 1964). Charles describes them as " fibrous stromal reaction ", " fibroblast-like stromal cells ", " a celular stroma which might be mistaken for endometrial sarcoma ". Comparing the steroid-induced tumours in mice and women one cannot avoid the impression that in the laboratory animal the sarcomatous trend is structurally much more pronounced than in women.

The structural difference of these tumours between women and mice is possibly due to the great difference in the duration of treatment. Our tumours of the endometrial stroma appeared exceptionally, in 1 case only, in a group of 31 animals treated for 13 months with 117 to 900 μg./day of progesterone; but their incidence increased to 11 out of 55 animals receiving 117 to 900 μg./day of progesterone during 18 months. The 18 months of mice correspond to 40 or 45 years in women! These chronological considerations may render more easy the understanding of the difference as to the pathological patterns elicited in the endometrial stroma by contraceptives, in women on one hand, and in our laboratory animals on the other.

As to the foci of cells, which are certainly different from those of the sarcoma originating in the endometrial stroma or around glands in endometriosis, classification is much more difficult than with sarcoma (compare Fig. 16 and 17 with Fig. 8 to 15). Glands or degenerated rests of glands are present in the respective foci both of the endometrial stroma (Fig. 16C) and of the space between the two muscular layers (Fig. 17B). This makes one suppose that these foci, similarly to the sarcoma, belong always to the endometrial stroma. Are they sarcomata in a process of evolution?

SUMMARY

A variety of pathological changes is elicited in the uterus by the prolonged administration of progesterone and 19-nor-contraceptives.

These pathological changes are: formation of cystic glands; endometriosis, including that of cystic glands and the appearance of the latter on the surface of the uterus; tumours of the endometrial stroma varying structurally between fibrosarcoma and sarcoma; metaplasia of the endometrial and glandular epithelium; foci of cells in the uterine wall possibly on the way to sarcoma.

The pathogenic activity of the steroids examined differs considerably: tumours of the endometrial stroma are produced by progesterone and norethindrone but not by norethynodrel. On the other hand norethynodrel produces metaplasia of the endometrial and glandular epithelium whereas progesterone and norethindrone are devoid of this action.

Results obtained with the prolonged administration of progesterone give evidence that the appearance of the various pathological patterns is fundamentally dependent both on the quantity of the steroid administrated and on the duration of the administration.

Most sincere thanks are due to Dr. Roberto Barahona, Professor of Pathology in the Faculty of Medicine of Universidad Católica de Chile, for advice as to uterine tumours; to the producers of steroids mentioned in our first two papers; to our technical and secretarial staff.

This study was aided by a grant from the Population Council, New York.

### REFERENCES

Charles, D.—(1964) *J. clin. Path.*, **17**, 205.
Lipschutz, A.—(1960) *Acta Un. int. Cancr.*, **19**, 149.
Lipschutz, A., Iglesias, R., Panasevich, V. I. and Salinas, S.—(1966) *Nature, Lond.*, **212**; 686.—(1967a) *Brit. J. Cancer*, **21**, 144, 153.
Okada, H., Amatsu, M., Ishihara, S. and Tokuda, G.—(1964) *Acta Endocr.*, **46**, 31.
Paulsen, A.—(1965) *Metabolism*, **14**, 313.
Meissner, A. and Sommers, S. C.—(1966) *Cancer Res.*, **26**, 474.